# Identification of the *RcbZIP* gene family in *Rubus Chingii* and analysis of the regulation of flavonoid synthesis by *RcbZIP1* and *RcbZIP12*

**DOI:** 10.3389/fpls.2026.1769751

**Published:** 2026-04-10

**Authors:** XiuRong Xu, JiuXin Lai, Shiming Cheng

**Affiliations:** Zhejiang Academy of Forestry, Hangzhou, Zhejiang, China

**Keywords:** expression analysis, functional analysis, gene family, identification, RcbZIP

## Abstract

*Rubus Chingii* is a multi-functional plant that can be used both as medicine and food. The fruit is rich in flavonoids, especially in their unripe fruits, where the content is even higher. This study identified and analyzed the members of the *bZIP* gene family and explored its regulatory mechanism on flavonoid synthesis. In total, 46 *RcbZIP* members were identified in this study. They were unevenly distributed across six chromosomes, with the largest number on chromosome 2, reaching 15. Subcellular localization predictions were all located in the nucleus. Based on the evolutionary tree analysis, the 46 members were divided into 13 subfamilies, and the motifs and gene structures of different subfamilies were highly consistent. Analysis of *cis*-acting elements revealed that their promoters are rich in light-responsive, abscisic acid-responsive, methyl jasmonate-responsive, and other elements. There are eight pairs of collinear genes in the genome, most of which have been purified and selected. WGD and TRD are the main reasons for the expansion of the *RcbZIP* gene family, and most members exhibit greater collinearity with dicotyledonous plants. Transcriptome analysis revealed that the two *RcbZIP*s (*RcbZIP1* and *RcbZIP12*), which had high expression levels when the fruit was ripe, but their expression levels decreased when the fruit was ripe. Further analysis revealed that *RcbZIP1* and *RcbZIP12* were significantly positively correlated with nine flavonol synthesis genes that were differentially expressed in unripe fruits. Notably, the FPKM values of *RcbZIP1* and *RcbZIP12* were significantly positively correlated with *LG05.2134(Rc4CL)*, the correlation coefficients of 0.83 for both. RT-qPCR confirmed that *RcbZIP1 and RcbZIP12* were highly expressed during the immature stage. Subcellular localization results showed that RcbZIP1 and RcbZIP12 were localized to the nucleus. Yeast monohybrids demonstrated that *RcbZIP1* and *RcbZIP12* can bind to the *LG05.2134(Rc4CL)* promoter, and LUC experiments proved that *RcbZIP1* and *RcbZIP12* can promote the expression of *LG05.2134(Rc4CL)*. This study lays the foundation for enhancing the medicinal value of *Rubus Chingii*.

## 1 Introduction

*Rubus Chingii* is a plant with both medicinal and edible uses that is widely distributed along the eastern coast of China (Hua et al., 2023). The medicinal value of *Rubus Chingii* is mainly attributed to its unripe fruits, whose medicinal effects are recorded in the Chinese Pharmacopoeia and have been demonstrated by modern pharmacological studies to possess antioxidant, anti-aging, and blood sugar-regulating properties (Sheng et al., 2020; Wu et al., 2024). Unripe *Rubus Chingii* fruits contain various components, such as flavonoids, terpenoids, and polysaccharides, among which flavonoids have been studied most extensively (Sheng et al., 2020; Wu et al., 2024; Xu et al., 2024; Shan et al., 2025). Flavonoids, including flavones, flavonols, anthocyanins, and isoflavones, have been extensively investigated by scientists because of their antioxidant, anticancer, and anti-inflammatory functions (Shen et al., 2022). In recent years, researchers have discovered many enzymes involved in the flavonoid biosynthesis pathway (Li P. et al., 2021). The main process of flavonoid synthesis is that phenylalanine is catalyzed by PAL (Phenylalanine Ammonialyase) to form cinnamic acid; cinnamic acid is catalyzed by C4H (Cinnamate 4-Hydroxylase) and 4CL (4-Coumarate CoA Ligase) to form coumaroyl-CoA; coumaroyl-CoA is catalyzed by CHS (Chalcone Synthease) to form chalcone; chalcone is transformed into naringenin by CHI (Chalcone Isomerase); naringenin is catalyzed by F3H (Flavonoid 3-Hydroxylase) to form dihydrokaempferol; dihydrokaempferol is catalyzed by F3’H (Flavonoid 3’-Hydroxylase) to form dihydroquercetin; dihydrokaempferol and dihydroquercetin can be catalyzed by FLS (Flavonol Synthase) to form flavonols or by DFR (Dihydroflavonol 4-Reductase) to form leucoanthocyanidins; leucoanthocyanidins are catalyzed by ANS (Anthocyanidin Synthase) to form anthocyanins (Deshmukh et al., 2018; Ni et al., 2020; Liu et al., 2022; Du et al., 2024). The genes encoding these enzymes are often highly expressed in unripe *Rubus Chingii* fruits and are significantly positively correlated with the content of most flavonoids (Hua et al., 2023). For example, LG07.1189 (RcCHI), LG01.2604 (RcF3H), LG05.396 (RcF3’H), LG02.1317 (RcFLS), LG04.4245 (RcCHS), LG04.4244 (RcCHS), LG06.4119 (RcPAL), LG07.48 (Rc4CL), and LG05.2134 (Rc4CL). These genes are often regulated by various transcription factors, such as MYB, bHLH, WRKY, and bZIP, which regulate the transcription of flavonoid synthase (Cuihua et al., 2024; Xu et al., 2026).

Basic leucine zipper (bZIP) proteins are a class of transcription factors that are widely involved in plant growth and development, as well as in biotic and abiotic stress responses. Their structure is relatively conserved, typically consisting of a DNA-binding basic region and a dimerizing leucine (Leu) zipper region (Rai et al., 2023). In recent years, an increasing number of bZIP transcription factors have been identified in plants. For instance, 78 *AtbZIP* transcription factors have been identified in *Arabidopsis thaliana* (Dröge-Laser et al., 2018), 56 *MabZIP* transcription factors in *Morus alba* (Liu et al., 2025a), 65 *PgbZIP* transcription factors in *Punica granatum* (Wang et al., 2022), 40 *GbbZIP* transcription factors in *Ginkgo biloba* (Han et al., 2021), and 65 *IibZIP* transcription factors in *Isatis indigotica* (Jiang et al., 2022). With the deepening of research, it has been discovered that bZIP transcription factors play a significant role in regulating flavonoid synthesis in plants. Overexpression of the bZIP transcription factors *JrHY5* and *GbbZIP08* in tobacco increased flavonoid content (Han et al., 2023; Zhang et al., 2025). In soybeans, *GmbZIP131* enhances flavonoid synthesis to resist salt stress, and *GmbZIP5* increases the content of isoflavones in hairy roots by binding to *GmMYB176* (Anguraj Vadivel et al., 2021; Ni et al., 2025). In grapes, both *VvbZIP36* and *VvbZIP22* promote the transcription of FLS, thereby increasing flavonoid content (Liu G. et al., 2025; Liu GC. et al., 2025).

Although bZIP transcription factors have been identified in many plants, no such identification has been made in *Rubus Chingii*. Moreover, there are no reports on whether *RcbZIP* transcription factors regulate flavonoid synthesis. Therefore, in this study, we identified all *RcbZIP* transcription factors based on the reported *Rubus Chingii* genome and annotation files. The physicochemical properties, phylogenetic tree, gene structure, and *cis*-acting elements in the promoter of the *RcbZIP* transcription factor family were analyzed using bioinformatics techniques. Simultaneously, based on transcriptome analysis, the RcbZIP family members most likely to regulate flavonoid synthesis were identified. RT-qPCR and dual-luciferase reporter gene experiments were conducted to confirm their potential in regulating the flavonoid synthesis pathway. This study lays a theoretical foundation for enhancing the medicinal value of *Rubus Chingii* through molecular biology.

## 2 Materials and methods

### 2.1 Identification of the *RcbZIP* gene family, analysis of the physicochemical properties of the proteins and construction of the evolutionary tree

The initial data preparation involved downloading the genome sequence and annotation file of *Rubus Chingii* from the Rosaceae genome database (https://www.rosaceae.org/) (Wang et al., 2021). The bZIP domain file with the Pfam database number PF00170 was downloaded from (http://pfam-legacy.xfam.org/), and all 78 AtbZIP protein sequences were downloaded from the TAIR database (https://www.arabidopsis.org/). Subsequently, the bZIP domain and all AtbZIP sequences were compared using the Simple HMM Search and Blast Several Sequences to a Big Database programs of the TBtools software, and the intersection of the comparison results was uploaded to the Interproscan database (https://www.ebi.ac.uk/interpro/result/interprosca/) for the final determination of all RcbZIP gene family members (Chen et al., 2023).

To better understand the basic information of the family members, this study analyzed the key physicochemical properties, such as amino acid quantity, molecular weight, isoelectric point, and instability coefficient, of the RcbZIP protein using the Expasy ProtParam online tool (https://web.expasy.org/protparam/). The position information of the family members was extracted using the GXF Gene Position and Info. Extract program of the TBtools software (Chen et al., 2023) was used. Finally, the specific location of the family member proteins in the cell was predicted using the BUSCA database (https://busca.biocomp.unibo.it/).

To better understand the grouping of family members, we constructed an evolutionary tree of *Rubus Chingii* and the bZIP family in *Arabidopsis thaliana*. First, all protein sequences were aligned using the MAFFT v7.471 program, then an evolutionary tree was constructed using MEGA7 software, and the tree was displayed on the Chiplot website (https://www.chiplot.online/) (Su et al., 2023). According to the grouping of the 78 AtbZIP protein sequences in the TAIR database (https://www.arabidopsis.org/), RcbZIP in *Rubus Chingii* was grouped.

### 2.2 Analysis of motifs and gene structure of the *RcbZIP* gene family

The MEME online tool (https://meme-suite.org/) was used to analyze the motifs in the RcbZIP protein sequence, resulting in the identification of 10 conserved motif sequences. These conserved motif sequences were uploaded to the InterProScan database (https://www.ebi.ac.uk/interpro/result/interprosca/) for functional annotation analysis. Finally, the Gene Structure View of the TBtools software was used to visualize the motifs, conserved domains, and gene structures (Chen et al., 2023).

### 2.3 Analysis of the *cis*-acting elements of the *RcbZIP* gene family

Using the Fasta Extract program of the TBtools software, the 2000bp promoter sequence located 2000 base pairs upstream of the ATG initiation codon of all *RcbZIP* gene family members was extracted. This sequence was then uploaded to the PlantCARE database (http://bioinformatics.psb.ugent.be/webtools/plantcare/) to predict *cis*-acting regulatory elements, and the number of such elements was counted. Finally, the results were presented as a heat map using the HeatMap program in TBtools (Chen et al., 2023).

### 2.4 The intragenic colinearity of the *RcbZIP* gene family in the genome

Collinearity analysis of the members of the *RcbZIP* gene family was conducted using the MCScanX software, and the collinearity diagram was drawn using the Advanced Circos function of the TBtools software. The Ka/Ks values (Nonsynonymous Substitution Rate/Synonymous Substitution Rate, non-synonymous Substitution rate/synonymous substitution Rate) of the collinearity gene pairs were calculated using the Simple Ka/Ks Calculator of the TBtools software (Wang et al., 2012; Chen et al., 2023). Replication types of the *RcbZIP* gene family were analyzed using DupGenfinder. Replication types were classified into five categories: whole-genome duplication (WGD), Transposed Duplication (TRD), Dispersed Duplication (DSD), Tandem Duplication (TD), and Proximal Duplication (PD) (Qiao et al., 2019).

### 2.5 Analysis of collinearity of the *RcbZIP* gene family among different species

The genome and annotation files of maize and grape were downloaded from the Chinese National Genome Database (https://ngdc.cncb.ac.cn/), the genome and annotation files of rice and *Arabidopsis thaliana* were downloaded from the Ensembl Plants database (https://plants.ensembl.org/), and the genome and annotation files of *Fragaria vesca*, *Malus domestica*, *Pyrus communis*, *Prunus persica*, *Rubus Idaeus*, and *Rubus occidentalis* were downloaded from the Rosaceae Genome Database (https://www.rosaceae.org/). MCScanX software was used to conduct a collinearity analysis of the members of the *RcbZIP* gene family with *bZIP* genes of different species. Finally, the results were presented as a heat map using the HeatMap program of the TBtools software (Wang et al., 2012; Chen et al., 2023).

### 2.6 Expression patterns of *RcbZIP* gene family and flavonoid synthesis gene family members during fruit ripening process

Transcriptome data of *Rubus Chingii* at different stages (mg, gy, yo, and re) were obtained from NCBI (bioproject number: PRJNA671545) (Li X. et al., 2021), and transcriptome data at different stages (GG, GY, YR, and RR) were also obtained (bioproject number: PRJNA1023155) (mg and GG are green fruits, gy and GY are yellow fruits, yo and YR are orange fruits, and re and RR are red fruits) (Hua et al., 2023). Transcriptome data were obtained using the Illumina sequencing platform, and the clean reads obtained from NCBI were aligned with the *Rubus Chingii* genome using Hisat2 v2.0.5 software. The reads of each gene were calculated using the FeatureCounts tool (http://subread.sourceforge.net/), and the Fragments Per Kilobase pair per million reads (FPKM) values were calculated based on the transcript length. The average values of the RcbZIP gene family in each period were calculated and presented as a heatmap using the HeatMap program of the TBtools software (Chen et al., 2023).

### 2.7 The expression patterns of the *RcbZIP* gene family and the flavonoid synthesis gene family members during fruit ripening process

The correlation between the *RcbZIP* gene family and flavonoid synthesis gene family members during fruit development was analyzed using the Chiplot website (https://www.chiplot.online/), and a correlation heatmap was drawn (Su et al., 2023).

### 2.8 RT-qPCR verification and subcellular localization

The *Rubus Chingii* fruits that had grown for 30, 40, 50, and 60 days were collected from the Forestry Academy of Zhejiang Province and frozen with liquid nitrogen for storage at -80°C for subsequent cloning of genes and qRT-PCR experiments. Extract RNA from young leaves using the Plant RNA Extraction Kit (Takara, Beijing, China), and test the purity of the RNA using the NanoPhotometer^®^ spectrophotometer (IMPLEN, CA, USA). The RNA was reverse transcribed into cDNA using the PrimeScriptTM RT reagent Kit with gDNA Eraser (Takara, Beijing, China). RT-qPCR analysis was performed on 12 samples (four periods of 30, 40, 50, and 60 days, with three replicates for each treatment, totaling 12 samples) using the SYBR PrimeScript RT-PCR Kit (Takara, Beijing, China). The ABI 7500 Real-Time PCR system (ABI 7500, Thermo Fisher, Singapore) was used. The relative expression levels of the genes were calculated using the 2^-ΔΔCT^ method (Livak and Schmittgen, 2001). Fluorescent quantitative PCR primers were designed using the Batch q-PCR Primer Design of TBtools software. The primer information is provided in **Supplementary Table S1**, where actin is the internal reference gene in *Rubus Chingii* (Chen et al., 2023). Finally, a bar chart was drawn using GraphPad Prism 10.

### 2.9 Dual luciferase reporter gene verification

Using seamless cloning technology, the ORF sequences of the *RcbZIP1* and *RcbZIP12* genes without stop codons were ligated to the pAN580 vector, and GFP was connected at the N-terminus. These recombinant vectors were then introduced into Arabidopsis protoplasts using the polyethylene glycol (PEG)-mediated transformation method. Under 470 nm excitation light, the GFP fluorescence signal was observed using a laser confocal microscope to analyze the localization of RcbZIP1 and RcbZIP12 proteins in cells (Yoo et al., 2007). The ORF sequences of the *RcbZIP1* and *RcbZIP12* genes were connected to the pGreen II 62-SK effect vector, and connect the 3000 bp upstream promoter sequence of the *LG05.2134(Rc4CL)* gene before the ATG start codon was connected to the upper end of the pGreen II 0800-LUC vector as a control. Both recombinant vectors were transformed into the Agrobacterium GV3101 strain of the root-knot fungus and introduced into tobacco protoplasts via Agrobacterium infection. Tobacco leaves were cultured in the dark for 24 h and then transferred to a 25 °C constant temperature incubator for alternating 16 h of light and 8 h of darkness for 2 d. The LUC fluorescence signal was observed using a chemiluminescence imaging system (Tanon, Tanon 5200, Shanghai, China), while the measurement of LUC and REN fluorescence activities was carried out using a dual luciferase reporter gene detection kit (Vazyme, DL101-01, Nanjing, China), and the relative ratio of LUC/REN was calculated.

### 2.10 Yeast single-hybrid experiment

The ORF sequences of *RcbZIP1* and *RcbZIP12* were ligated to the pGADT effector vector, and the 3000 bp promoter sequence upstream of the ATG start codon of the *LG05.2134(Rc4CL)* gene was ligated to the pAbAi reporter vector.The auto-activation test revealed that 100 ng/mL of Aureobasidin A (AbA) could effectively inhibit yeast growth. Finally, both the recombinant vectors were transformed into the YIH Gold cells, and their interaction was assessed based on yeast growth on an Synthetic Dropout - Uracil and Leucine selection medium supplemented with 100 ng/mL AbA.

## 3 Results

### 3.1 Identification of the *RcbZIP* gene family, analysis of protein physicochemical properties, and construction of the evolutionary tree

**Figure 1:**
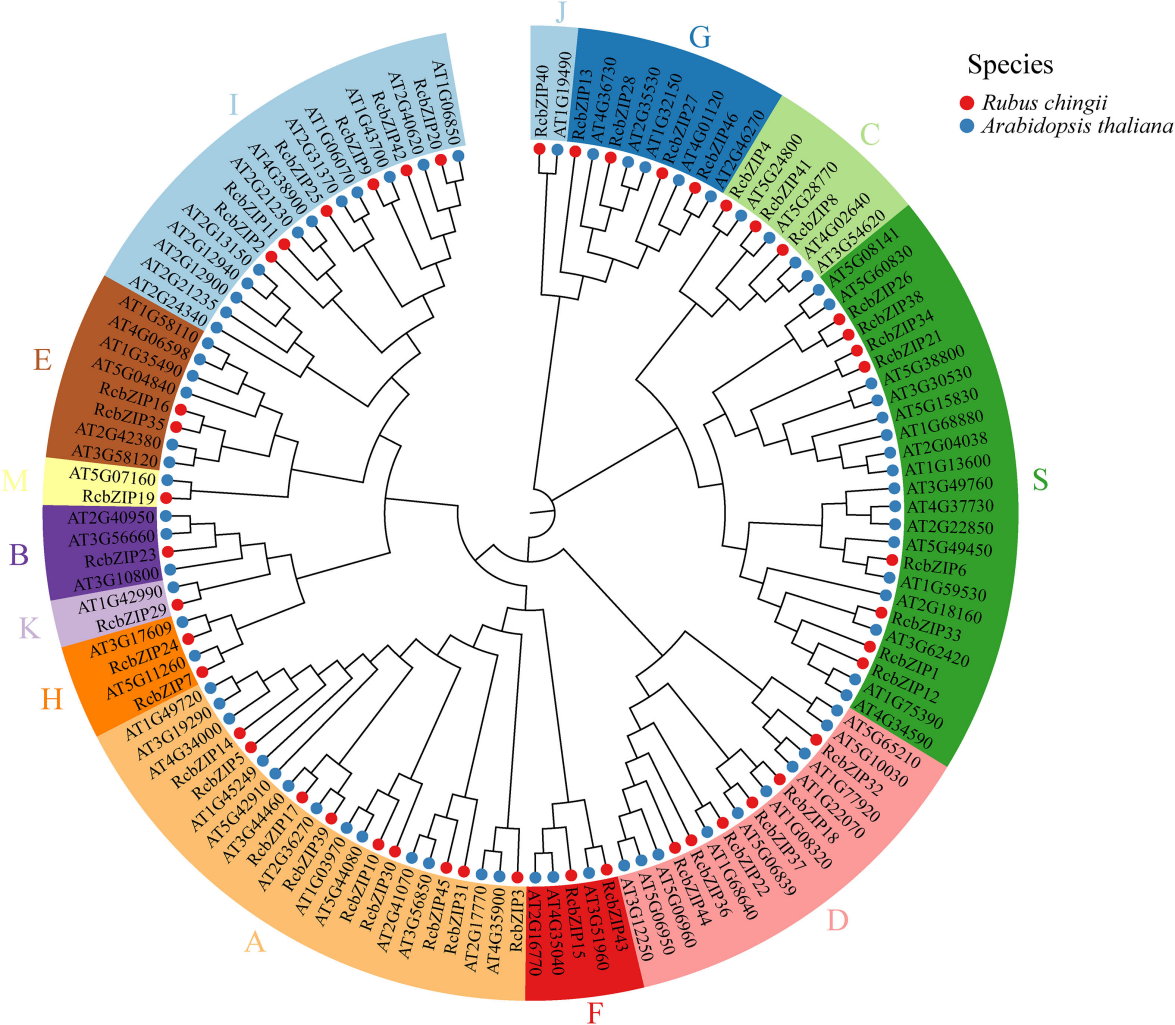
Phylogenetic tree based on the amino acid sequences of *Rubus Chingii* and *Arabidopsis thaliana*. Different colors of gene names indicate different subfamilies. At, *Arabidopsis thaliana*; Rc, Rubus Chingii.

A total of 46 *RcbZIP* gene family members were identified in *Rubus Chingii*. Chromosomal location analysis revealed that 15 members of *RcbZIP*s were located on chromosome 2, 5 members of *RcbZIP*s were located on chromosome 3, 5 members of *RcbZIP*s were located on chromosome 4, 8 members of *RcbZIP*s were located on chromosome 5, 8 members of *RcbZIP*s were located on chromosome 6, and 5 members of *RcbZIP*s were located on chromosome 7, and no *RcbZIP*s were found on chromosome 1 (**Supplementary Table S2**). The members were named RcbZIP1-RcbZIP46 according to their distribution on the chromosomes. The physicochemical properties of the 46 RcbZIPs were analyzed, and the number of amino acids ranged from 146 to 705 (aa), molecular weights ranged from 17303.42 (Da) to 76122.2 (Da), isoelectric points ranged from 4.24 to 10.06, instability coefficients ranged from 34.89 to 73.49, and average hydrophobicity ranged from -1.317 to -0.453 (**Supplementary Table S2**). Subcellular localization prediction showed that all 46 RcbZIP proteins were located in the nucleus (**Supplementary Table S2**). To explore the evolutionary relationships of the 46 RcbZIPs in *Rubus Chingii*, a phylogenetic tree was constructed by combining the 46 RcbZIPs of *Rubus Chingii* with 78 AtbZIP proteins of *Arabidopsis thaliana*. Phylogenetic analysis revealed that 124 bZIP proteins were unevenly distributed across 13 subfamilies. Among them, subfamily A contained the largest number of members, with 22 members in total, including nine RcbZIPs and 13 AtbZIPs. The B subfamily had four members, including one RcbZIP and three AtbZIPs. The C subfamily had seven members, including three RcbZIPs and four AtbZIPs. The D subfamily contained 16 members, including six RcbZIPs and 10 AtbZIPs. The E subfamily had eight members, including two RcbZIPs and six AtbZIPs. The F subfamily had five members, including two RcbZIPs and three AtbZIPs. The G subfamily had nine members, including four RcbZIPs and five AtbZIPs. The H subfamily contained four members, including two RcbZIPs and two AtbZIPs. The I subfamily contained 18 members, including six RcbZIPs and 12 AtbZIPs. The J subfamily had two members, including one RcbZIP and one AtbZIP. The K subfamily had two members, including one RcbZIP and one AtbZIP. The M subfamily had two members, including one RcbZIP and one AtbZIP. The S subfamily contained 25 members, including eight RcbZIPs and 17 AtbZIPs (**Figure 1**).

### 3.2 Analysis of motif distribution and gene structure of the *RcbZIP* gene family

**Figure 2:**
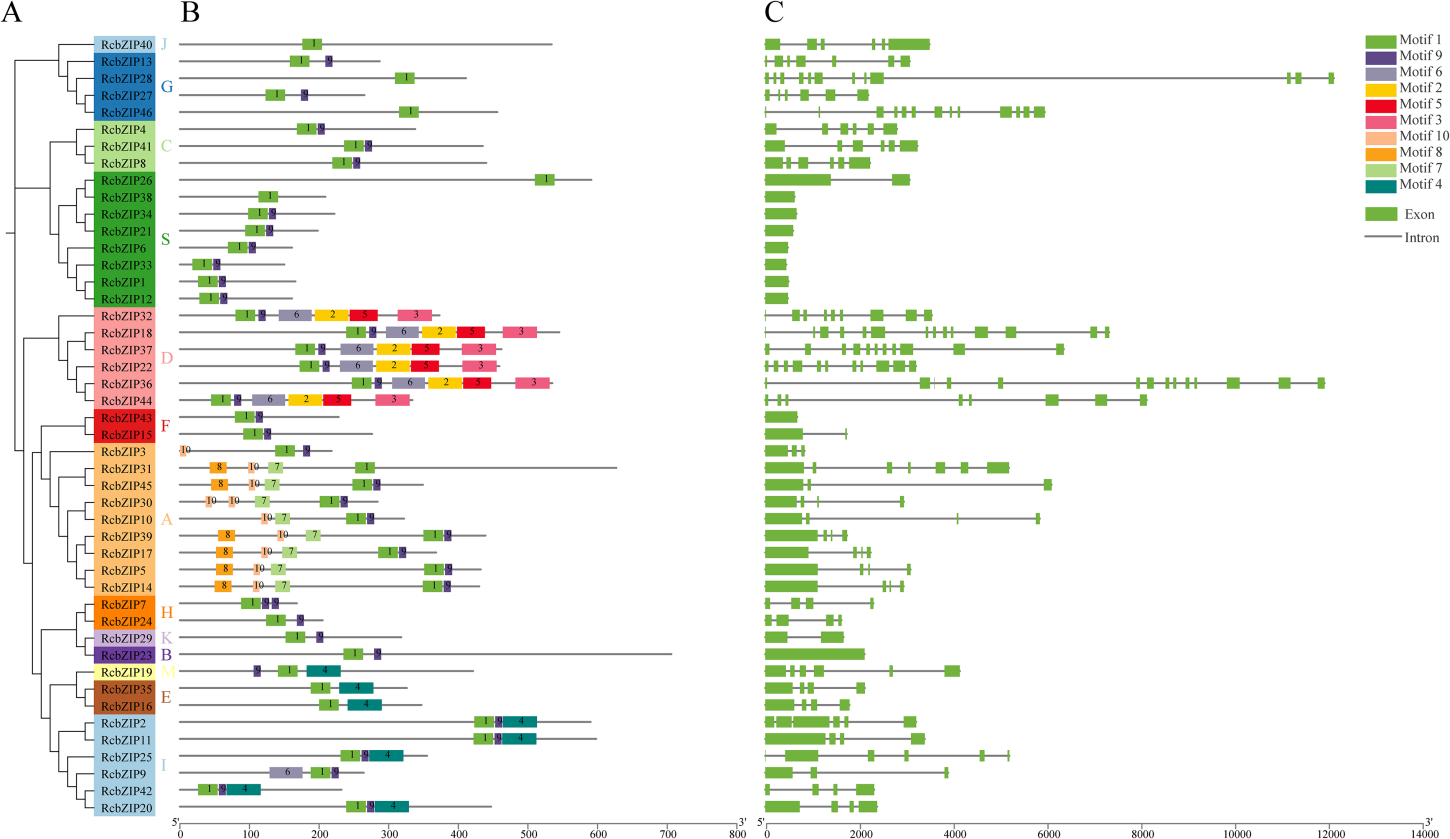
Motif and gene structure analysis. **(A)**The evolutionary tree of the *RcbZIP* family **(B)** The ten conserved motifs in RcbZIPs. Conserved motifs of the RcbZIPs were identified using the online MEME program based on full-length amino acid sequences with the following parameters: maximum number of motifs, 10; maximum width, 100. The lengths and positions of different motifs in the protein sequences are identified by the lengths and positions of the different color blocks. **(C)** Gene structure of *RcbZIP*s. Exons, introns, and untranslated regions (UTRs) are indicated by green rectangles, black lines, and black rectangles, respectively.

In this study, we identified the distribution of the top 10 conserved motifs in the *RcbZIP* gene family. The analysis results showed that the motif sequences contained by the gene family members in different subfamilies exhibited a high degree of consistency, and some motifs were specific to certain subfamilies, such as Motifs 2, 3, and 5 being present only in the D subfamily members, and Motifs 7 and 10 being present only in the A group (**Figures 2A, B**). Additionally, the analysis revealed that Motif 1 was a conserved sequence in the bZIP domains (**Supplementary Table S3**). Further gene structure analysis revealed that the gene structures of members within the same subfamily were highly consistent; for example, the number of exons in the S subfamily members was at least 1-2 (**Figure 2C**).

### 3.3 Statistical analysis of the number of *cis*-regulatory elements in the *RcbZIP* gene family

**Figure 3:**
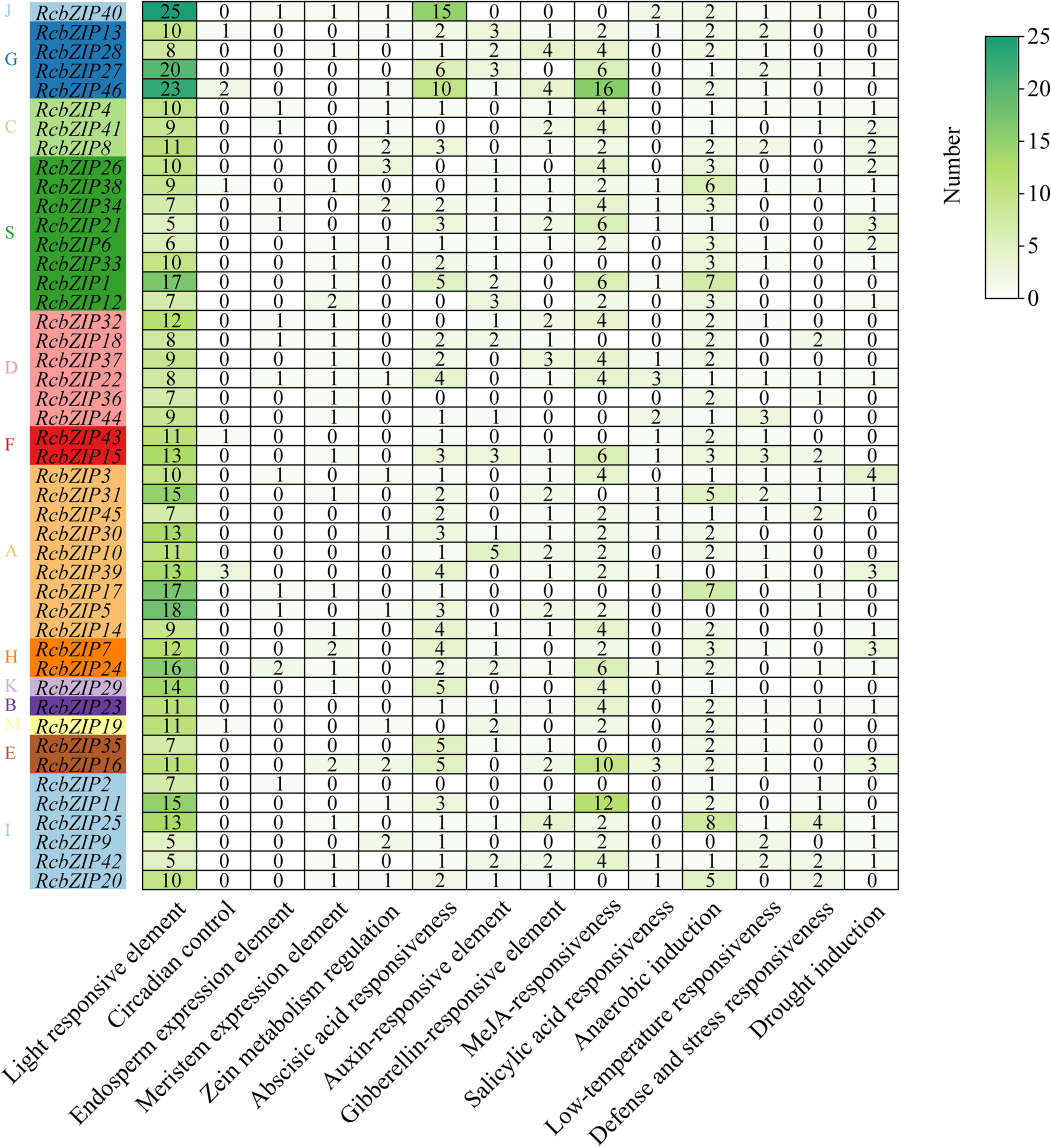
*Cis-* elements in the promoter regions of *RcbZIPs.* The colored block with a number represents the *cis*-element number of *RcbZIPs*.

Analysis of the cis-acting elements revealed that the promoters of the members of the RcbZIP gene family are rich in various cis-acting elements, mainly including response elements related to growth development, hormones, and stress conditions (**Figure 3**). Among these elements, those related to growth development were the most abundant, with light response elements being the most abundant. The most common hormone response elements were abscisic acid and jasmonic acid methyl ester response elements, and the most common stress response elements were anaerobic induction and drought response elements. The heatmap clearly shows that, except for the light response elements, the most abundant cis-acting elements of the RcbZIP gene family are the abscisic acid response and jasmonic acid methyl ester response elements.

### 3.4 Co-linearity analysis of the RcbZIP gene family and analysis of gene duplication types

**Figure 4:**
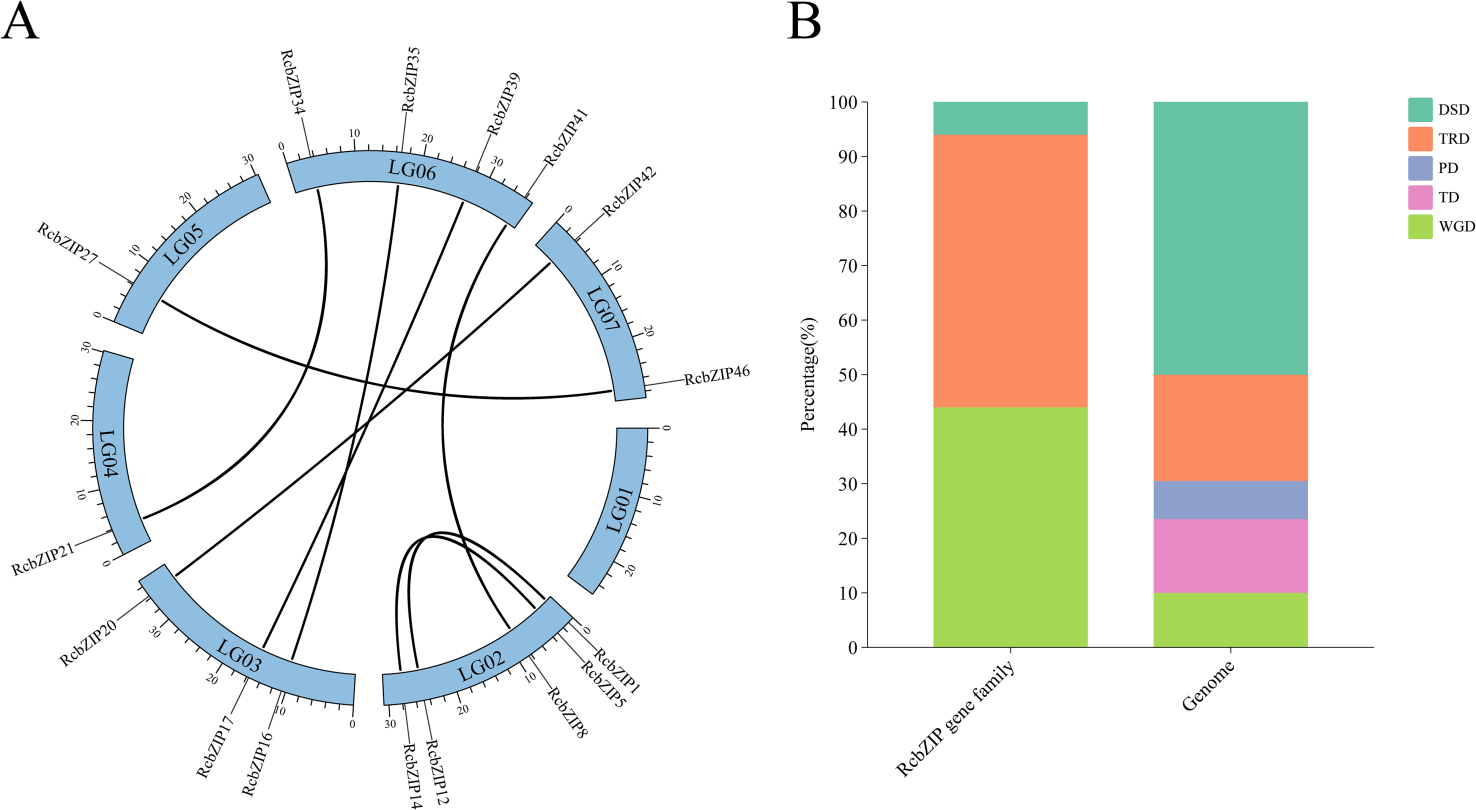
**(A)** Intra-genomic collinearity of the *RcbZIP* genes. **(B)** Duplication types of the *RcbZIP* genes. TRD, DSD, PD, TD and WGD indicate transposon duplication, dispersed duplication, proximal duplication, tandem duplication and whole genome duplication in the figure.

A co-linearity analysis was conducted within the *Rubus Chingii* genome, and eight pairs of co-linearity gene pairs were identified (**Figure 4A**). Further calculations of their Ka/Ks values revealed that, except for the three pairs of genes (*RcbZIP16*/*RcbZIP35*, *RcbZIP17*/*RcbZIP39*, and *RcbZIP21*/*RcbZIP34*) that showed significant divergence, the Ka/Ks values of the other gene pairs were all less than 1 (**Supplementary Table S4**). Further analysis revealed eight pairs of WGD, one pair of DSD, and nine pairs of TRD in the *RcbZIP* gene family (**Supplementary Table S5**). Compared to the entire genome, WGD and TRD played a much greater role in the expansion of the *RcbZIP* gene family (**Figure 4B**).

### 3.5 Homology of the RcbZIP gene family with other species

**Figure 5:**
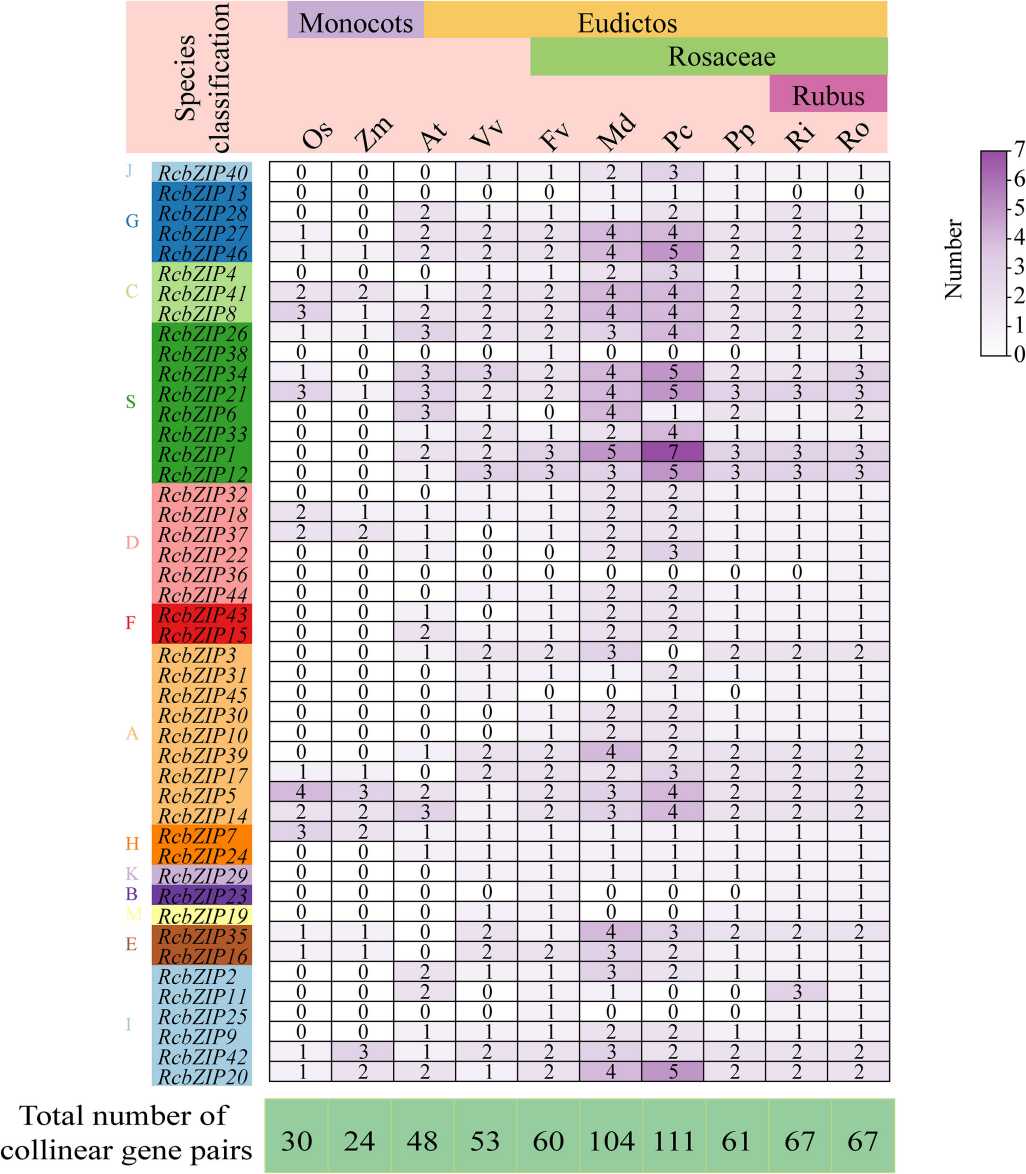
The number statistics of orthologous gene pairs in the *RcbZIP* gene family compared with other species. Os, *Oryza sativa*; Zm, *Zea mays*; At, *Arabidopsis thaliana*; Vv, *Vitis vinifera*; Fv, *Fragaria vesc*; Md, *Malus domestica*; pc, *Pyrus communis*; pp, *Prunus persica*; Ri, *Rubus Idaeus*; Ro, *Rubus occidentalis*; Among them, *Oryza sativa* and *Zea mays* are monocots plants, while the others are eudictos plants. Among the eudictos plants, except for Arabidopsis and grape, the rest belong to the Rosaceae family.

Through the analysis of the homology of the *RcbZIP* gene family with other species, it was found that there are 30 pairs of homologous genes with rice, 24 pairs with corn, 48 pairs with *Arabidopsis thaliana*, 53 pairs with grape, 60 pairs with *Fragaria vesca*, 104 pairs with *Malus domestica*, 111 pairs with *Pyrus communis*, 61 pairs with *Prunus persica*, 67 pairs with *Rubus Idaeus*, and 67 pairs with *Rubus occidentalis* (**Figure 5**). Further analysis revealed that a few members of most families were homologous with monocotyledonous plants, whereas most members were homologous with dicotyledonous species. For example, members of the J, F, B, and M subfamilies only showed homology with dicotyledonous plants (**Figure 5**).

### 3.6 Expression patterns of the RcbZIP gene family during fruit ripening and correlation analysis with flavonoid synthesis genes

**Figure 6:**
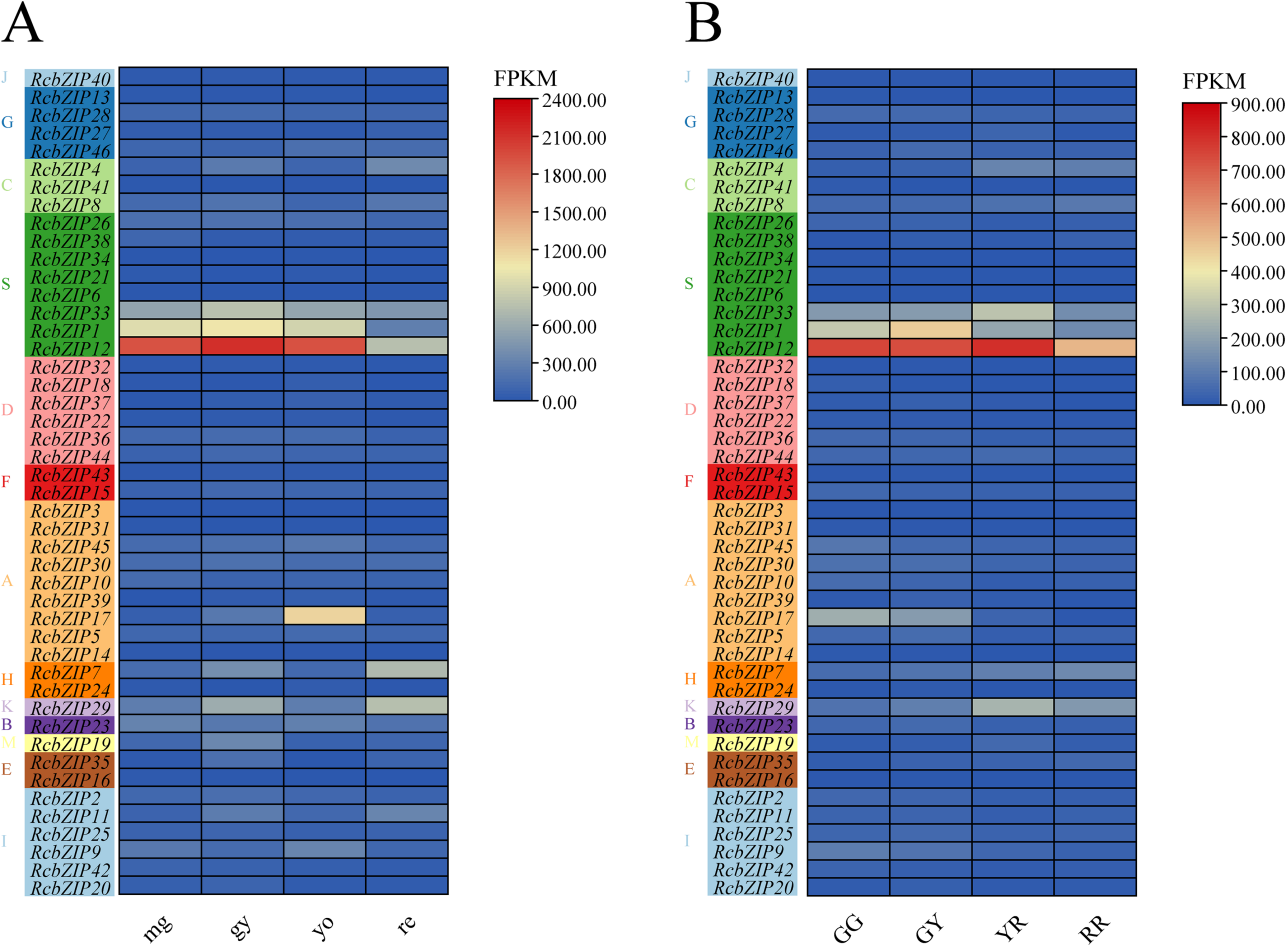
The expression pattern of *RcbZIP* genes. **(A)** The expression patterns of the *RcbZIP* gene family in different periods of mg, gy, yo and re during fruit ripening, **(B)** The expression patterns of the *RcbZIP* gene family in different periods of gg, gy, yr and rr during fruit ripening.

**Figure 7:**
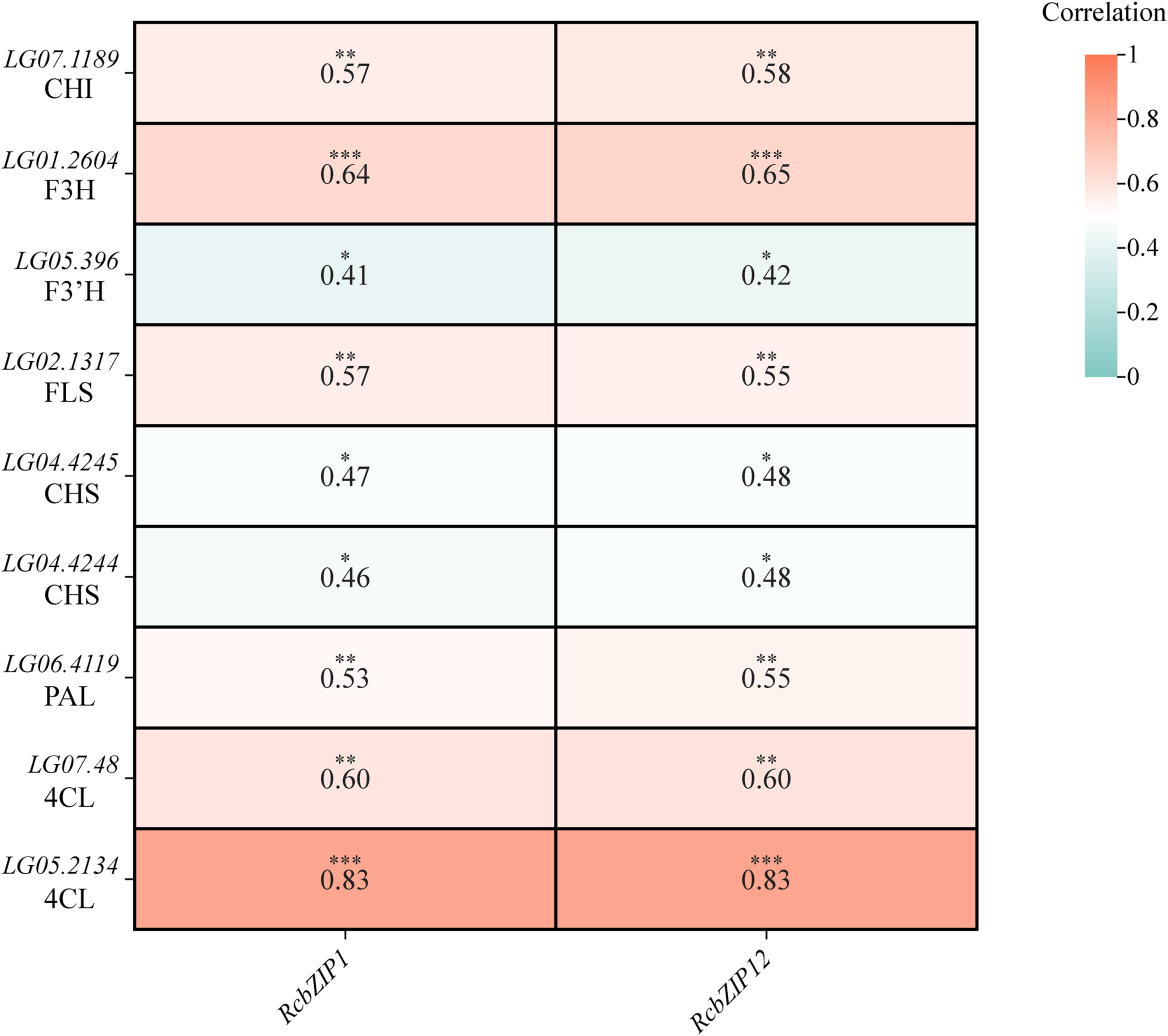
Heatmap showing the correlation between the expression levels of flavonoid synthesis genes that are highly differentially expressed in the immature state of *Rubus Chingii* and the expression level of *RcbZIP* gene. The stars represent significance; one star indicates P < 0.05 in the student’s t-test, two stars indicate P < 0.01 in the student’s t-test, and three stars indicate P < 0.001 in the student’s t-test.

The transcriptional group FPKM value heatmap clearly shows that, except for a few members, the expression levels of most members were extremely low during the fruit ripening process. By comparing **Figure 6A** with **Figure 6B**, it can be observed that the expression levels of *RcbZIP1* and *RcbZIP12* are higher before fruit ripening, whereas they significantly decrease after fruit ripening. Furthermore, correlation analysis with previously studied flavonoid synthesis genes that were highly differentially expressed during fruit ripening revealed that *RcbZIP1* and *RcbZIP12* were significantly positively correlated with the expression levels of these genes. The key gene for regulating flavonoid synthesis, *LG05.2134* (*Rc4CL*) was correlated with a coefficient of 0.83 with the FPKM values of *RcbZIP1* and *RcbZIP12* expression levels (**Figure 7**).

### 3.7 RT-qPCR verification, subcellular localization verification, and dual luciferase reporter gene verification

**Figure 8:**
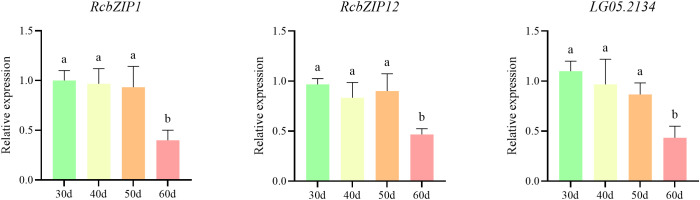
Relative expression levels of *RcbZIP1*, *RcbZIP12* and *LG05.2134(Rc4CL)*. The letters above the bars indicate significant differences, as determined by the student’s t-test.

**Figure 9:**
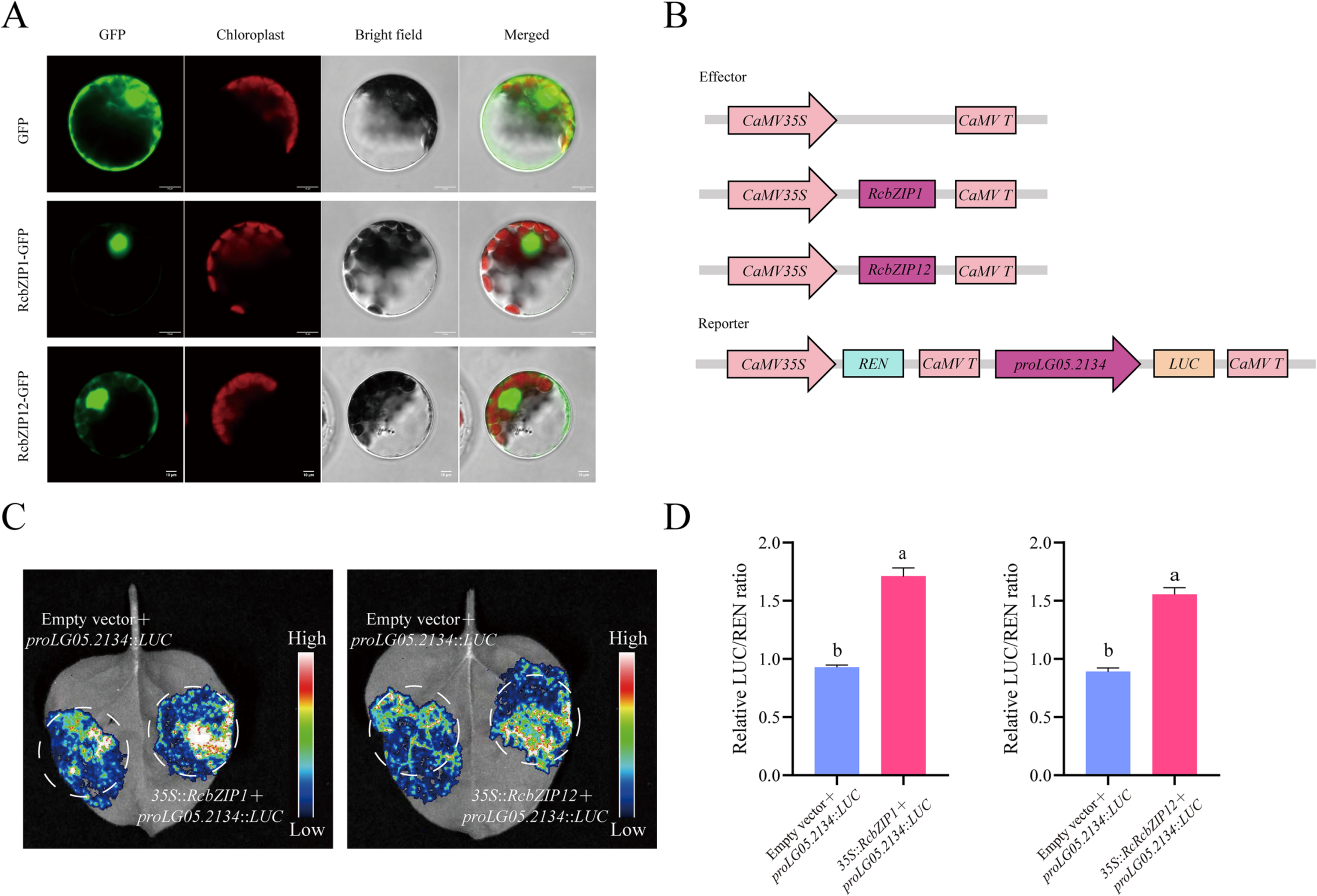
**(A)** Subcellular localizations of *RcbZIP1* and *RcbZIP12*. **(B)** Constructs used in the LUC assay. **(C)** Intensity of luciferase activity in leaves. **(D)** Relative LUC/REN fluorescence intensity. The letters above the bars indicate significant differences, as determined by the student’s t-test.

To verify the reliability of the transcriptome data, we conducted RT-qPCR on *RcbZIP1*, *RcbZIP12*, and *LG05.2134(Rc4CL)*. The results showed that *RcbZIP1*, *RcbZIP12*, and *LG05.2134(Rc4CL)* were significantly highly expressed during the fruit unripening period (**Figure 8**). The subcellular localization results indicated that both RcbZIP1 and RcbZIP12 were located in the nucleus, consistent with the predicted results (**Figure 9A**). Based on **Figure 9B**, an expression vector was constructed, and *RcbZIP1* and *RcbZIP12* were selected as effector genes, while *proLG05.2134(Rc4CL)* was selected as the reporter promoter to verify the regulation of *Rubus Chingii* flavonoid synthesis by the *RcbZIP* gene family. The fluorescence intensity was significantly higher in leaves co-transfected with 35S:: RcbZIP1 or 35S:: RcbZIP12 than in those transfected with *proLG05.2134(Rc4CL)*::LUC alone (**Figure 9C**). Quantitative detection also confirmed that the Luiferase activity was stronger in the leaves co-transfected with 35S::RcbZIP1 or 35S::RcbZIP12, indicating that both *RcbZIP1* and *RcbZIP12* could specifically bind to *proLG05.2134(Rc4CL)* and exert positive regulatory effects (**Figure 9D**).

### 3.8 Yeast one-hybrid

**Figure 10:**
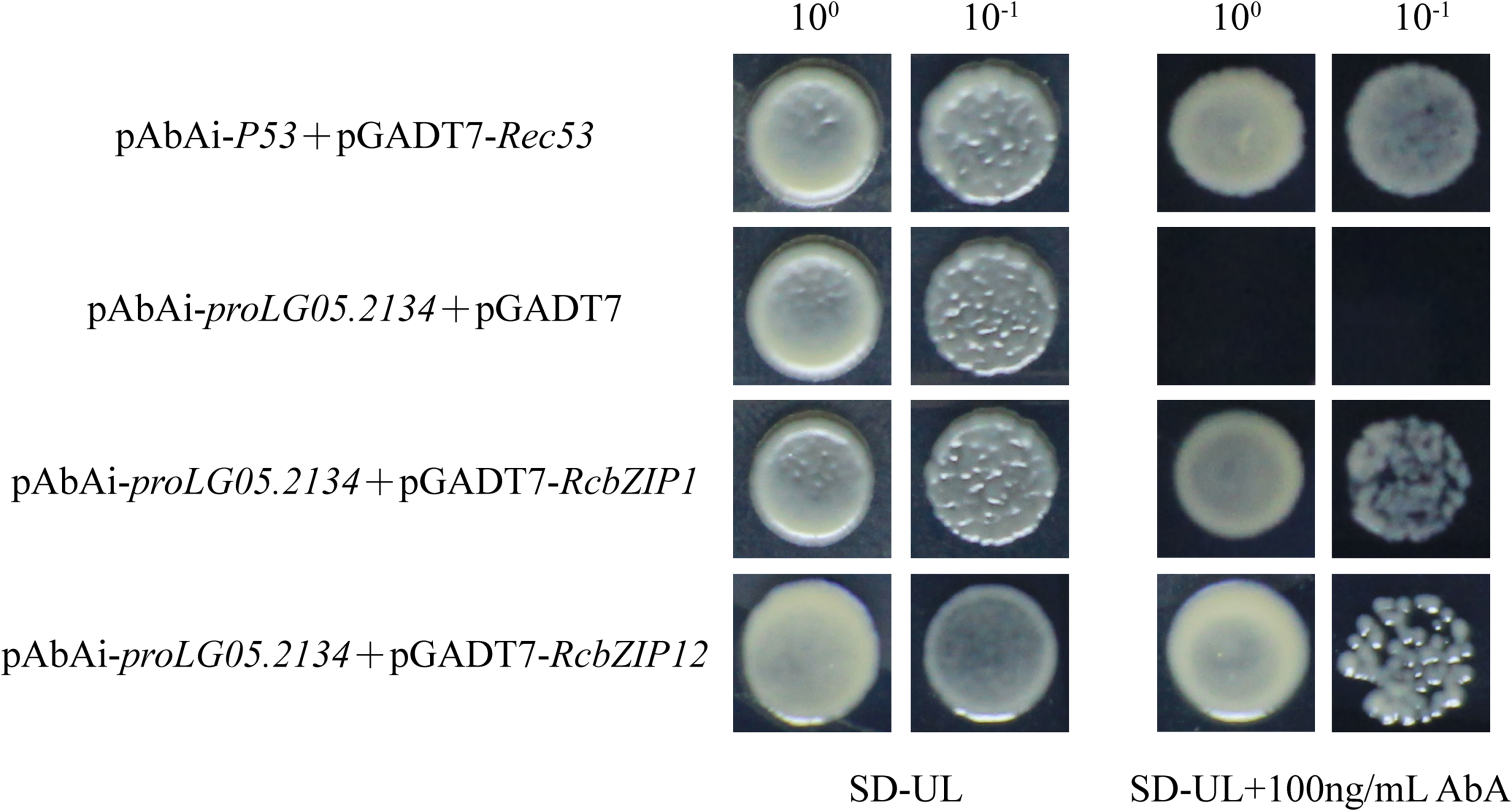
Growth of yeast transformed with diferent vectors. Growth of yeast cells carrying both *proLG05.2134(Rc4CL)*-AbAi and pGAD7-*RcbZIP1*(*RcbZIP12*) vectors indicates activation of the reporter gene. Here, 10^0^, and 10^-1^ represent the diution factors of the yeast solution used in the assay, and SD-ULepresents SD medium lacking uracil (URA) and leucine (LEU).

Further, the effector gene and reporter vector were constructed and transformed intoY1H Gold yeast strains to verify the interaction between the two.The experimental group grows on the medium containing AbA, it indicates that the corresponding transcription factor (*RcbZIP1* or *RcbZIPT12*) can bind to the promoter *proLG05.2134(Rc4CL)* and activate the expression of the reporter gene (**Figure 10**).

## 4 Discussion

The *bZIP* gene family has been identified in multiple species, and numerous studies have investigated its crucial role in regulating plant flavonoid synthesis (Zhou et al., 2023). To better understand the *bZIP* gene family in *Rubus Chingii*, this study identified 46 members of the *RcbZIP* gene family based on the *Rubus Chingii* genome. Previous studies have found that the number of *bZIP* gene family members in 12 species of the Rosaceae family ranges from 44 to 121, indicating that the overall number of *bZIP* members in *Rubus Chingii* is relatively small (Zhu L. et al., 2024). The subcellular prediction and experimental results both confirmed that the bZIP protein is located in the nucleus, which is consistent with previous research results (Yin et al., 2024; Yang H. et al., 2025). By constructing a phylogenetic tree with *Arabidopsis thaliana*, we found that compared to the various subfamilies of *Arabidopsis thaliana*, the members of the *Rubus Chingii* subfamilies, except for a few subfamilies, are fewer than those of *Arabidopsis thaliana* (Dröge-Laser et al., 2018). Simultaneously, the protein motifs of each subfamily were highly consistent with the gene structure, which is consistent with the results of most previous studies on bZIP gene families and also proves the conservation of the bZIP gene family in plants (Liang et al., 2022; Wang et al., 2024). The heatmap of cis-regulatory elements shows that most *cis*-regulatory elements on the promoters of RcbZIP gene family members are light response elements, abscisic acid response elements, and jasmonic acid methyl ester response elements. These results are consistent with studies on the promoters of bZIP gene families in Neopyropia yezoensis, Liriodendron chinense, and Stevia rebaudiana (Li et al., 2022; Wu M. et al., 2023; Zhu X. et al., 2024). In Artemisia annua, the *AabZIP* gene was found to be differentially expressed in response to different light conditions (Rai et al., 2023). Additionally, many experiments have confirmed that *bZIP* genes are highly expressed under abscisic acid and jasmonic acid methyl ester treatments (Wu X. et al., 2023; Jha et al., 2024). These results suggest that members of the *RcbZIP* gene family may function in response to different light inductions, abscisic acid, and jasmonic acid methyl ester.

Gene duplication is the main mechanism for generating new genes and ultimately for generating new biological functions, and it is crucial for plant evolution and adaptation to the environment. Genomic collinearity is an important method for studying gene duplication (Qu et al., 2023). In the *Rubus Chingii* genome, there are only eight pairs of collinear gene pairs of the *RcbZIP* gene family, which is fewer than that of other species in the Rosaceae family. This explains why the number of *RcbZIP* gene family members in the *Rubus Chingii* genome is less than that of these Rosaceae species (Zhu L. et al., 2024). Except for the three pairs of genes, *RcbZIP16*/*RcbZIP35*, *RcbZIP17*/*RcbZIP39*, and *RcbZIP21*/*RcbZIP34*, which are highly divergent, the Ka/Ks values of the other gene pairs are all less than 1, indicating that most members are undergoing purifying selection pressure after gene duplication (Yan et al., 2022; Wu et al., 2025). The collinearity between species can be used to understand the formation time of gene family members (Yang Y. et al., 2025). A few members of the *RcbZIP* gene family showed collinearity with monocot plants, while most members showed collinearity with dicot species, indicating that the formation time of most members was after the differentiation of monocot and dicot plants. This conclusion has also been found in the *CmbZIP* gene family of chestnut (Zhang et al., 2023).

As an important fruit that can be used both as medicine and food, the flavonoid content of *Rubus Chingii* has always been a focus of attention. This study specifically focused on the expression pattern of the *RcbZIP* gene in *Rubus Chingii* fruits from the immature to the mature stage, attempting to identify the key *RcbZIP* transcription factor that regulates the flavonoid content in *Rubus Chingii* fruits. The results showed that The FPKM values of the *RcbZIP1* and *RcbZIP12* genes were higher in unripe fruits and significantly decreased after the fruits matured. Previous studies have found that the flavonoid content in *Rubus Chingii* fruits is much higher in green, yellow, and orange fruits than in red fruits during the unripe period. This suggests that *RcbZIP1* and *RcbZIP12* are likely key genes regulating flavonoid content in *Rubus Chingii* fruits (Li X. et al., 2021; Hua et al., 2023). Interestingly, both RcbZIP1 and RcbZIP12 belong to the S subfamily. Previous studies have also found that members of the bZIP protein S family have certain effects on the regulation of flavonoid substances (Liu et al., 2025a). Additionally, a large number of previous studies have shown that bZIP transcription factors play an important role in regulating the flavonoid content during fruit ripening, such as in pear, where *PpbZIP44* increases the flavonoid content in pear fruits during ripening (Wang et al., 2023), and in tomato and red pear, where bZIP-type transcription factors *SlHY5* and *PyHY5* can promote the accumulation of anthocyanins in tomato fruits (Qiu et al., 2019; Wang et al., 2025), and in apple, where *MdbZIP44* can promote the accumulation of anthocyanins together with *MdMYB1* (An et al., 2018). The C subfamily member *VvibZIPC22* is involved in the synthesis of flavonols in grape fruits. Overexpression of this gene in tobacco can significantly increase several types of flavonoids. Transient promoter experiments have demonstrated that *VvibZIPC22* may directly bind to the ACGT-containing elements in the promoter regions of *FLS1*, *ANR*, *CHS3*, and *CHI*, thereby activating the expression of related target genes (Malacarne et al., 2016). Further analysis of the transcriptome data revealed that *RcbZIP1* and *RcbZIP12* were significantly positively correlated with a large number of genes related to flavonoid synthesis, including *LG07.1189* (*RcCHI*), *LG01.2604* (*RcF3H*), *LG05.396* (*RcF3’H*), *LG02.1317* (*RcFLS*), *LG04.4245* (*RcCHS*), *LG04.4244* (*RcCHS*), *LG06.4119* (*RcPAL*), *LG07.48* (*Rc4CL*), and *LG05.2134* (*Rc4CL*) (Hua et al., 2023). This is consistent with the conclusions of previous extensive studies that bZIP transcription factors can promote the expression of flavonoid synthesis genes. This study further selected the *LG05.2134(Rc4CL)* gene, which had the highest correlation with *RcbZIP1* and *RcbZIP12*, for RT-qPCR verification. RT-qPCR confirmed that *RcbZIP1*, *RcbZIP12*, and *LG05.2134(Rc4CL)* were highly expressed in unripe fruits and had the lowest relative expression in red fruits at the 60-day stage. Previous studies have found that bZIP transcription factors can regulate the expression of the *4CL* gene. For example, in dandelions, the bZIP-type transcription factor *TmHY5* can respond to blue light induction and bind to the *Tm4C*L promoter region to promote its expression (Liu et al., 2025b), and in Salvia miltiorrhiza, the bZIP-type transcription factor *SmAREB1* can promote the expression of *Sm4CL1* (Jia et al., 2017). Therefore, the LUC test confirmed that both *RcbZIP1* and *RcbZIP12* specifically bind to *proLG05.2134(Rc4CL)* of the *4CL* gene family and exert positive regulatory effects.

The evidence supporting the regulation of flavonoid synthesis by RcbZIP1 and RcbZIP12, based solely on transcript level changes, remains preliminary. To further elucidate their functions, future studies will employ functional validation approaches such as heterologous expression in model systems.

## 5 Conclusion

In this study, a total of 46 members of the RcbZIP gene family were identified. The phylogenetic tree divided them into 13 subfamilies, and each subfamily showed similarities in motifs and gene structure. Further analysis revealed that most members have collinearity relationships in dicotyledonous plants, suggesting that the functions of RcbZIP gene family members may be similar to those of bZIP gene family members in other species. Transcriptome analysis revealed that RcbZIP1 and RcbZIP12 in the S family were highly expressed during the immature fruit stage. Members of the S subfamily in which they are located have the ability to regulate flavonoid synthesis, thus being considered as playing an important role in regulating flavonoid substances in Rubus Chingii fruits. Further correlation analysis also proved that RcbZIP1 and RcbZIP12 have significant correlations with flavonoid synthesis genes in Rubus Chingii fruits. Finally, RT-qPCR, LUC and yeast single-hybrid experiments verified that RcbZIP1 and RcbZIP12 were relatively highly expressed in immature Rubus Chingii fruits, and RcbZIP1 and RcbZIP12 could bind to the proLG05.2134(Rc4CL) gene of the 4CL gene family in tobacco leaves and promote downstream gene transcription. In the future, further in-depth exploration will be conducted on the mechanism by which RcbZIP1 and RcbZIP12 regulate the flavonoid content in Rubus Chingii fruits.

## Data Availability

The original contributions presented in the study are included in the article/**Supplementary Material**. Further inquiries can be directed to the corresponding author.

## Supplementary material

Supplementary Table 1 Primer sequences for qRT-PCR.

Supplementary Table 2 Protein physicochemical properties and subcellular localization prediction.

Supplementary Table 3 Motif sequence.

Supplementary Table 4 The Ka, Ks, Ka/Ks values of gene pairs identified by McScanX.

Supplementary Table 5 Protein duplication types of gene pairs.
